# Letter to the Editor Regarding “Assessment of Retinal Nerve Fiber Layer Thickness in Non-Diabetic Obese Children and Adolescents”

**DOI:** 10.4274/jcrpe.5205

**Published:** 2018-02-26

**Authors:** Ömer Karti

**Affiliations:** 1 University of Health Sciences, Bozyaka Training and Research Hospital, Clinic of Ophthalmology, İzmir, Turkey

**Keywords:** Obese children, optical coherence tomography, retinal nerve fiber layer

**To the Editor,**

I read the manuscript entitled “Assessment of Retinal Nerve Fiber Layer Thickness in Non-Diabetic Obese Children and Adolescents” recently published online by Ozen et al (1) in the J Clin Res Pediatr Endocrinol with great interest. I would like to thank Ozen et al (1) for their comments on our publication entitled “The assessment of peripapillary retinal nerve fiber layer and macular ganglion cell layer changes in obese children: a cross sectional study using optical coherence tomography” (2). I would like to clarify some of the misunderstandings raised by Ozen et al (1). They said that our manuscript included patients with high refractive status (up to 5 D) and that this situation could have affected retinal nerve fiber layer (RNFL) values by optical coherence tomography (OCT). However, in our study, both the study group and the control group had rather low refraction values. Our study demonstrated that spherical equivalent was -0.04±0.61 D in the obese group and -0.05±0.53 in the non-obese group. Although not mentioned in the paper, patients’ spherical equivalents ranged from +2.0 to -1.5 D. We completely agree with the authors that the presence of a refraction error can cause inaccurate measurement of OCT and RNFL values. The measurement errors of OCT parameters due to differences in axial length or refractive error causing ocular magnification effects have been documented by previous studies (3,4,5). OCT scans are typically angular. Hence, a 20 degree projection on a longer eye covers a larger area than on a hyperopic eye. The difference in scanned region (magnification), and the path used to quantify thickness is what causes differences in thickness versus refractive error or axial length.The transverse mirror in OCT is calibrated for an axial length of 24.46 mm. Inter-individual differences in axial length which vary from 24.46 mm would result in magnification errors in the measurements made on OCT (3,5,6). Therefore, to remove the effect of ocular magnification, the clinicians have to adjust the RNFL results using Littmann’s method (3,4,5,6,7). In this regard, the data present actual RNFL thickness in high refractive error.
